# Diagnostic and prognostic implications of bacteremia in patients with complicated pleural infection

**DOI:** 10.1515/pp-2023-0044

**Published:** 2024-03-01

**Authors:** Chang Ho Kim, Ji Eun Park, Jung Guen Cha, Jae Kwang Lim, Jongmin Park, Yong Hoon Lee, Sun Ha Choi, Hyewon Seo, Seung Soo Yoo, Shin Yup Lee, Seung Ick Cha, Jae Yong Park, Jaehee Lee

**Affiliations:** Department of Internal Medicine, School of Medicine, 65672 Kyungpook National University, Daegu, Republic of Korea; Department of Radiology, School of Medicine, 65672 Kyungpook National University, Daegu, Republic of Korea

**Keywords:** pleural infection, empyema, bacteremia, outcome, microbiology

## Abstract

**Objectives:**

The clinical significance of bacteremia in patients with complicated pleural infection is still uncertain. We aimed to examine the incidence and clinical significance of bacteremia in patients with complicated pleural infection.

**Methods:**

This retrospective study comprised of consecutive patients who received pleural drainage due to complicated parapneumonic effusion or empyema. The clinical, laboratory, and radiologic data and clinical outcome were compared between patients with and without bacteremia. Additionally, the factors associated with overall mortality were evaluated in these patients.

**Results:**

Of 341 patients included in the analysis, 25 (7 %) had a positive blood culture. Blood culture testing added 2 % identification of causative pathogen compared to pleural fluid culture alone. By multivariable analysis, radiologic features of cavitary lesion, a RAPID score≥5, and a positive microbial culture in pleural fluid were independently associated with bacteremia. Despite these clinical distinctions, there was ultimately no significant difference in in-hospital mortality between patients with and without bacteremia (3 vs. 4 %, p=1.0). The only factor significantly associated with overall mortality among patients with complicated pleural infections was a higher RAPID score [HR=1.96 (95 % CI=1.35–2.84)].

**Conclusions:**

The rate of bacteremia in patients with complicated pleural infection was 7 %. Blood culture testing demonstrated limited diagnostic yield and had minimal impact on clinical outcomes compared to pleural fluid culture. Therefore, it seems that blood culture testing is more advantageous for specific patients with suspected pleural infection who have cavitary lesions or a RAPID score≥5.

## Introduction

Pleural infections typically co-occur with pneumonia, although there exist alternative pathways for bacterial invasion into the pleural space, including hematogenous dissemination from systemic infection or transdiaphragmatic spread of intra-abdominal infection [1]. Globally, there has been a notable increase in the occurrence of pleural infections, and its short-term mortality rate varies from 4 % to over 26 % [2], [3], [4], [5], [6], [7], [8], [9], [10]. Therefore, it is evident that early diagnosis and prompt treatment are crucial in managing pleural infections.

The primary treatment modalities for complicated pleural infection, such as complicated parapneumonic effusions or empyema, consist of appropriate antibiotic selection and pleural drainage [1]. Knowledge of the causative pathogen in pleural infection is a crucial factor in determining the appropriate antibiotic regimen. Nonetheless, positive pleural fluid culture, which identifies the microbial etiology, is only present in 18–60 % of patients diagnosed with pleural infection [2, 7, 11, 12]. In addition, previous studies have demonstrated that the typical microbial causes of pleural infection differ from those of pneumonia [13, 14]. Thus, in more than half of the patients diagnosed with pleural infection, empirical antimicrobial treatment is given, which could result in uncertain treatment outcomes.

Bacteremia was present in 2–7% of patients diagnosed with pneumonia [15], [16], [17]. While blood culture was previously suggested as a routine diagnostic measure, it is currently reserved for those with severe pneumonia, as it has a low diagnostic yield and no prognostic impact on clinical outcomes [18]. The usefulness of blood cultures in pleural infection cases has not been extensively studied, despite the British Thoracic Society’s 2010 recommendation to obtain blood cultures from all suspected case [19]. The presence of bacteremia can have two different clinical implications. Firstly, it can be a prognostic factor associated with severe infections that can lead to a variety of clinical manifestations, such as sepsis, endocarditis, meningitis, and other serious infections [20, 21]. Secondly, it can provide valuable information about the susceptibility of the causative pathogen to antibiotics, which is essential for selecting appropriate antibiotics. Therefore, there is a need for further research to investigate the diagnostic and prognostic implications of blood culture testing in cases of pleural infection. This study aimed to examine the incidence and clinical significance of bacteremia and its impact on clinical outcomes in patients with complicated parapneumonic effusion or empyema who underwent percutaneous pleural drainage.

## Materials and methods

### Study population

We conducted a retrospective study on patients who had received pleural drainage due to complicated parapneumonic effusion or empyema at Kyungpook National University Hospital, a tertiary referral hospital in South Korea, which included consecutive cases between January 2011 and May 2021. Patients who were enrolled had either a complicated parapneumonic effusion, which was defined as a pleural fluid pH of less than 7.2, glucose less than 40 mg/dL, lactate dehydrogenase (LDH) greater than 1,000 U/L, or positive gram stain or culture or an empyema, defined by the presence of frank pus, based on the Light’s classification [22]. Those who did not undergo pleural drainage were excluded. Only the first occurrence of pleural infection was considered for patients with recurrent episodes.

### Data collection and definitions

Our institution’s standard protocol for patients suspected with pleural infection is to perform pleural fluid smear and cultures including pairs of aerobic and anaerobic blood culture bottles to detect aerobic and anaerobic microbial pathogens, mycobacteria, and fungi as well as two sets of blood cultures [11]. Pleural infection due to a nonbacterial pathogen such as fungus or mycobacterium was excluded in this study. Bacteremia was defined when there was the identification of the same organism in the blood culture and other cultures obtained from the pleural fluid/sputum or the isolation of the organism from two or more blood cultures drawn at different times. Except for methicillin-resistant *Staphylococcus aureus*, multidrug resistance (MDR) was defined as the lack of susceptibility to at least one agent in three or more categories of antimicrobial agents in drug sensitivity testing [23]. The standard initial treatment regimen involved the administration of empirical antibiotics and percutaneous catheter drainage, with or without antifibrinolytic therapy (urokinase or tissue plasminogen activator) given at the physician’s discretion. Details on data regarding demographics, comorbidities, infection acquisition site (community vs. hospital), blood and pleural fluid testing results, causative pathogens, and clinical outcome were collected from electronic medical record. RAPID score (Renal [urea], age, fluid purulence, infection source, and dietary [albumin]) was also calculated [24]. The data on in-hospital and post-discharge mortality, as well as the last-follow-up date, were collected.

A retrospective evaluation of radiological findings was conducted by a thoracic radiologist and a pulmonary specialist, regarding the amount of pleural effusion and loculation status of the effusion as seen on chest radiographs, and the presence of cavitary lesions observed on chest computed tomography scans. The clinical, laboratory, and radiologic data and outcome were compared between the two groups with and without bacteremia. The study protocols were approved by the Institutional Review Board of Kyungpook National University Hospital. Informed consent was waved due to the retrospective nature of the study.

### Statistical analysis

The statistical analyses were conducted utilizing IBM SPSS Statistics Version 22.0 for Windows (IBM Corp., Armonk, NY, USA). For the comparison of continuous variables, which were expressed as median and interquartile range (IQR), either the t-test or the Mann-Whitney U test was used, whereas the chi-square or Fisher’s exact test was utilized for comparing categorical variables, which were presented as number and percentage. Univariable and multivariable logistic regression analyses were performed to identify the factors associated with bacteremia in patients with complicated pleural infection, and the resulting odds ratio (OR) and 95 % confidence intervals (CI) were reported. Additionally, the Cox-proportional hazard regression analysis was employed to assess the risk factors associated with overall survival. Variables with p-values<0.05 were considered statistically significant.

## Results

### Comparisons of clinical, laboratory, and radiologic findings between complicated pleural infection patients with and without bacteremia

In this study, 341 patients with complicated pleural infections who received pleural drainage were included. Among them, 25 (7 %) had a positive blood culture. The median age for patients with bacteremia and those without bacteremia was 68 and 65 years, respectively (Table 1). In both groups, approximately 80 % of the patients were male. In total, 23 % had comorbid diabetes mellitus. Malignancy was observed to be more prevalent in patients with bacteremia than in those without (16 vs. 6 %, p=0.087). There was no significant difference in the occurrence of pleural infection due to extrapulmonary infection between the two groups. On the other hand, there was a tendency for hospital-acquired infections to occur more frequently in patients with bacteremia (24 vs. 13 %, p=0.133).

**Table 1:** Clinical, laboratory, and radiologic characteristics of patients with complicated pleural infection according to the presence or absence of bacteremia.

Variable	Total (n=341)	With bacteremia (n=25)	Without bacteremia (n=316)	p-Value
Age, years	65 (55–76)	68 (62–75)	65 (54–76)	0.172
Male	280 (82)	21 (84)	259 (82)	1.0
Ever-smoker	236 (69)	14 (56)	222 (70)	0.137
Underlying diseases				
Malignancy	24 (7)	4 (16)	20 (6)	0.087
Diabetes mellitus	77 (23)	6 (24)	71 (23)	0.860
Advanced liver disease	16 (5)	3 (12)	13 (4)	0.103
Chronic lung disease	24 (7)	0 (0)	24 (8)	0.238
Primary infection site				0.217
Lung	329 (97)	23 (92)	306 (97)	
Extra-pulmonary site	12 (3)	2 (8)	10 (3)	
Hospital acquired infection	47 (14)	6 (24)	41 (13)	0.133
Blood				
WBC count, cells/μL	14,160 (10,445–19,490)	12,620 (9,535–15,525)	14,425 (10,522–19,815)	0.350
CRP, mg/dL	20.5 (12.6–28.3)	21.8 (12.1–30.2)	20.5 (12.6–28.2)	0.473
BUN, mg/dL	15.7 (11.5–23.4)	23.5 (15.5–44.7)	15.4 (11.2–22.7)	<0.001
Albumin, g/dL	3.0 (2.6–3.5)	2.7 (2.4–3.2)	3.1 (2.6–3.5)	0.005
LDH, U/L	195 (164–247)	273 (191–406)	193 (162–237)	0.006
RAPID score	3 (2–4)	5 (4–5)	3 (2–4)	<0.001
Pleural fluid analysis				
WBC count, cells/μL	4,082 (1,192–15,263)	3,700 (1,425–24,534)	4,118 (1,107–14,994)	0.153
pH	7.19 (6.93–7.34)	7.19 (6.89–7.34)	7.20 (6.93–7.34)	0.166
Protein, g/dL	4.5 (3.9–5.0)	4.0 (3.6–4.4)	4.6 (4.0–5.0)	0.004
Glucose, mg/dL	73 (20–108)	100 (36–156)	71 (20–108)	0.626
LDH, U/L	1,236 (760–2,423)	1,706 (1,135–3,647)	1,198 (717–2,259)	0.002
Positive culture	119 (35)	17 (68)	102 (32)	<0.001
Empyema	47 (14)	4 (16)	43 (14)	0.762
Radiological findings				
Cavitary lesion	25 (7)	5 (20)	20 (6)	0.027
Large amount effusion^a^	61 (18)	5 (20)	56 (18)	0.787
Loculated effusion	81 (24)	10 (40)	71 (23)	0.047

Data are expressed as the median (interquartile range) or number (%). WBC, white blood cells; CRP, C-reactive protein; BUN, blood urea nitrogen; LDH, lactate dehydrogenase; RAPID, Renal (urea)/Age/fluid Purulence/Infection source/Dietary (albumin). ^a^Defined as effusion of more than two thirds of one hemithorax.

Patients with bacteremia had significantly lower levels of serum albumin and higher levels of blood urea nitrogen (BUN) and serum LDH on presentation compared to those without bacteremia (albumin: 2.7 [2.4–3.2] vs. 3.1 [2.6–3.5] g/dL, p=0.005; BUN: 23.5 [15.5–44.7] vs. 15.4 [11.2–22.7] mg/dL, p<0.001; LDH: 273 [191–406] vs. 193 [162–237] U/L, p=0.006). The RAPID score was significantly higher in patients with bacteremia than in those without bacteremia (5 [4–5] vs. 3 [2–4], p<0.001).

The analysis of pleural fluid revealed that patients with bacteremia had elevated levels of LDH and reduced levels of protein. In addition, patients with bacteremia had a significantly higher rate of positive pleural fluid culture results (68 vs. 32 %, p<0.001), with a two-fold increase in occurrence compared to those without bacteremia. Furthermore, patients with bacteremia exhibited a higher occurrence of cavitary lesions and loculated pleural effusion on radiological evaluation, compared to those without bacteremia (20 vs. 6 % for cavitary lesions, p=0.027; and 40 vs. 23 % for loculated effusion, p=0.047).

Multivariable analysis showed that cavitary lesion (OR=3.88 [95 % CI=1.20–12.52], p=0.023), RAPID score≥5 (OR=2.70 [95 % CI=1.12–6.51], p=0.027), and pleural fluid culture positivity (OR=4.40 [95 % CI=1.75–11.03], p=0.002) were the independent factors associated with bacteremia in patients with complicated pleural infection.

### Identification of microbial etiologies from the blood and pleural fluid cultures in patients with complicated pleural infection

Among 341 patients with pleural infection, 127 (37 %) had the causative pathogens identified through blood (n=25, 7 %) or pleural fluid culture (n=119, 35 %) (Table 2). Eight among 25 patients with bacteremia showed no growth in pleural fluid culture, accounting for 2 % of the total study population and 6 % of the population with identified pathogens. The causative pathogens from the blood culture included *Staphylococcus* species (36 %; *S. aureus* [n=8] and *Staphylococcus hemolyticus* [n=1]), *Streptococcus* species (32 %; *Streptococcus pneumoniae* [n=3], *Streptococcus pyogenes* [n=2], *Streptococcus sanguinis* [n=1], *Streptococcus parasanguinis* [n=1], and *Streptococcus salivarius* [n=1]), *Klebsiella pneumoniae* (20 %, n=5), and others (12 %; *Escherichia coli* [n=2], and *Enterococcus fascium* [n=1]). Among 17 patients with complicated pleural infection with both blood and pleural fluid culture-positive results, 14 yielded consistent organisms across blood and pleural fluid cultures, while three had different organisms in blood and pleural fluid cultures (Supplementary Table 1). Polymicrobial infections and MDR-pathogens were identified in nine (7 %) and 40 (32 %) out of 127 patients with a positive blood or pleural fluid culture, respectively. The proportion of MDR pathogens was higher in patients with bacteremia than in those without bacteremia (52 vs. 27 %, p=0.014). Drug susceptibility testing results for 14 isolates from both the blood and pleural fluid simultaneously were consistent between blood and pleural fluid.

**Table 2:** Results of blood culture as compared with pleural fluid culture.

	Pleural fluid culture (+)	Pleural fluid culture (−)	Total
Blood culture (+)	17 (5)	8 (2)	25 (7)
Blood culture (−)	102 (30)	214 (63)	316 (93)
Total	119 (35)	222 (65)	341 (100)

On the other hand, among the 102 patients who tested positive for pleural fluid culture but did not have bacteremia, the primary causative pathogens were identified as *viridans streptococci* species (n=54, 53 %), *K. pneumoniae* (n=24, 24 %), and *Staphylococcus* species (n=10, 10 %).

### Comparisons of clinical outcomes between complicated pleural infection patients with and without bacteremia

Approximately 50 % of patients received intrapleural fibrinolytic therapy, which was similar between patients with and without bacteremia (Table 3). The length of pleural catheter drainage was significantly longer in patients with bacteremia, with a median of 14 days (7–23) compared to 8 days (5–13) in those without bacteremia (p=0.003). However, there were no significant differences in the number of patients who underwent decortication operation or experienced in-hospital mortality between the two groups (decortication operation: 8 vs. 3 %, p=0.217; in-hospital mortality: 3 vs. 4 %, p=1.0). The in-hospital mortality rates for the groups with positive blood cultures alone, positive pleural fluid cultures alone, positive cultures in both blood and pleural fluid, and negative cultures in both were 0 , 6.9, 7.7, and 2.3 %, respectively.

**Table 3:** Clinical outcomes of patients with complicated pleural infection according to the blood culture results.

Variable	Total (n=341)	With bacteremia (n=25)	Without bacteremia (n=316)	p-Value
Intrapleural fibrinolytic therapy	177 (52)	11 (43)	166 (53)	0.411
Time to PCD removal, d	8 (5–14)	14 (7–23)	8 (5–13)	0.003
Decortication operation	12 (4)	2 (8)	10 (3)	0.217
In-hospital mortality	13 (4)	1 (4)	12 (4)	1.0

Data are expressed as the median (interquartile range) or number (%). PCD, percutaneous catheter drainage.

In patients with complicated pleural infection, several factors were found to be associated with overall mortality according to univariable Cox proportional hazard regression analysis. These factors included old age, advanced liver disease, hospital acquired infection, serum albumin levels, pleural fluid culture positivity, and RAPID score. However, in multivariable analysis, only the RAPID score was significantly associated with overall mortality (hazard ratio [HR]=1.96 [95 % CI=1.35–2.84], p<0.001) (Table 4). Pleural fluid culture positivity showed an increased tendency for overall mortality (HR=2.25 [95 % CI=0.86–5.88], p=0.098).

**Table 4:** Univariable and multivariable Cox-proportional hazard regression analyses for factors associated with overall mortality of patients with complicated pleural infection.

	Univariable analysis		Multivariable analysis	
Variable	HR (95 % CIs)	p-Value	HR (95 % CIs)	p-Value
Age, years	1.07 (1.02–1.11)	0.004		
Male	0.56 (0.20–1.58)	0.277		
Malignancy	2.86 (0.83–9.88)	0.097		
Diabetes mellitus	1.26 (0.45–3.54)	0.660		
Advanced liver disease	4.22 (1.22–14.66)	0.023		
Hospital acquired infection	4.94 (1.90–12.80)	0.001		
WBC count, cells/μL	1.00 (1.00–1.00)	0.749		
Serum CRP, mg/dL	0.96 (0.91–1.00)	0.074		
Serum albumin, g/dL	0.36 (0.19–0.67)	<0.001		
Serum LDH, U/L	1.00 (0.99–1.01)	0.888		
RAPID score	2.07 (1.43–2.99)	<0.001	1.96 (1.35–2.84)	<0.001
PF pH	1.45 (0.27–7.84)	0.664		
PF protein	0.69 (0.47–1.01)	0.056		
PF glucose, mg/dL	1.00 (0.99–1.01)	0.333		
PF LDH, U/L	0.86 (0.58–1.27)	0.445		
Empyema	1.28 (0.37–4.43)	0.694		
Cavitary lesion	1.21 (0.16–9.11)	0.853		
Large amount effusion^a^	1.03 (0.29–3.55)	0.968		
Loculated effusion	0.51 (0.20–1.32)	0.164		
Positive blood culture	1.59 (0.37–6.94)	0.534		
Positive PF culture	3.03 (1.17–7.81)	0.022	2.25 (0.86–5.88)	0.098
MDR pathogen	1.99 (0.66–6.07)	0.222		

HR, hazard ratio; CIs, confidence intervals; WBC, white blood cells; CRP, C-reactive protein; LDH, lactate dehydrogenase; RAPID, Renal (urea)/Age/fluid Purulence/Infection source/Dietary (albumin); PF, pleural fluid; MDR, multi-drug resistant. ^a^Defined as effusion of more than two thirds of one hemithorax.

## Discussion

The present study revealed that 7 % of patients with complicated pleural infections requiring pleural drainage yielded a positive blood culture result. Compared to relying on pleural fluid culture testing alone, blood culture testing provided an additional 2 % identification of the causative pathogen in cases of pleural infection. Blood culture positivity was further associated with RAPID score≥5, radiologically cavitary lesion, and pleural fluid culture positivity. Despite this, a positive blood culture result did not exhibit an association with mortality, although patients with bacteremic complicated pleural infection displayed a prolonged duration of pleural drainage compared to their non-bacteremic counterparts. The only factor significantly associated with overall mortality among patients with complicated pleural infections was a higher RAPID score.

This study’s observation of the rate of positive blood cultures in cases of pleural infection aligns with earlier researches indicating a blood culture positivity rate of 2–12 % among patients with pleural infections [2, 25, 26]. The 2010 recommendation from the British Thoracic Society suggests obtaining blood cultures from all suspected cases of pleural infection based on the MIST1 study [2, 19]. The study found that positive blood culture results were often observed in patients with no other positive microbiology results although the number of such cases was not reported [2]. However, in the current study, only 2 % of patients diagnosed with complicated pleural infection had positive blood culture results despite negative pleural fluid culture results. Therefore, performing blood cultures selectively on patients with pleural infection, similar to the approach used for pneumonia [18, 27], might be more appropriate. However, there was no available data regarding the factors associated with blood culture positivity in patients with complicated pleural infection. This study showed that a RAPID score≥5 or the presence of a cavitary lesion could serve as appropriate clinical indications for pre-treatment blood cultures in patients with pleural infection.

In addition to the diagnostic significance of blood culture testing, the influence of bacteremia on clinical outcomes is a crucial factor to consider when determining the necessity of blood culture testing in patients with pleural infection. The RAPID scoring system, which takes into account BUN, age, purulence, infection source, and albumin levels, is considered to be the primary prognostic indicators for pleural infection [24]. This study found that a higher RAPID score was significantly linked to overall mortality in patients with complicated pleural infections, consistent with previous research [9, 24, 28]. On the other hand, the presence of bacteremia did not seem to have a substantial impact on mortality in these patients, consistent with other recent studies suggesting that bacteremia was not associated with an increased risk of mortality in patients with pneumococcal pneumonia, including those admitted to intensive care unit [27, 29]. The relatively lower diagnostic and prognostic value of blood culture testing implies limited usefulness in patients with pleural infection.

The mortality rate of pleural infection in this study was 4 %, which is relatively lower compared to the previously reported range of 4–26 % [2], [3], [4], [5], [6], [7], [8], [9], [10]. These results may be partially attributed to the fact that the mortality rate was found to be relatively high in cases of hospital-acquired pleural infection [7, 13], while 86 % of patients in this study had community-acquired pleural infection. Moreover, the mortality rate could be impacted by the virulence of the infecting organism. This study included a lower number of *S. aureus* or Gram-negative bacilli, which are known to be associated with higher mortality [1, 13, 25]. Finally, the mortality rate of the study population may be affected by the proportion of empyema cases, given its relatively high mortality rate, which ranges from 11-26 % [9, 10]. However, it should be acknowledged that the number of cases with empyema in this study was relatively small.

Several limitations need to be addressed in this study. Firstly, the retrospective design may suffer from bias and provide limited control over the quality and completeness of the data collected. Secondly, the study’s single-center design may restrict the generalizability of the findings to other settings. Furthermore, the small sample size, with only 25 patients having positive blood cultures, may limit the study’s statistical power. This study did not assess the role of urinary pneumococcal antigen testing in patients with pleural infection due to the unavailability of data for all individuals in the study population. Lastly, the study did not report information on the timing, type, and duration of antibiotic treatment, which could have influenced outcomes such as mortality. However, the single-center design of the study may have facilitated the implementation of more uniform treatment protocols, and the relatively low in-hospital mortality rate observed among our patients could be a reflection of such management, which closely adheres to standard treatment practices.

In conclusion, this study found that among patients with complicated pleural infection requiring pleural drainage, 7 % had bacteremia, which was associated with a RAPID score≥5, cavitary lesion, and pleural fluid microbial culture positivity. Blood culture testing showed limited additional diagnostic value as well as there was no significant difference in mortality rates between patients with and without bacteremia. A larger population study is further required for the reevaluation of the value of blood culture testing in patients with pleural infection.
